# Complex treatment of oily polluted waters by modified melamine foams: from colloidal emulsions to a free oil removal

**DOI:** 10.1007/s11356-023-29055-x

**Published:** 2023-08-21

**Authors:** Sarah Hailan, Patrik Sobolciak, Anton Popelka, Peter Kasak, Samer Adham, Igor Krupa

**Affiliations:** 1 Center for Advanced Materials, Qatar University, P. O. Box 2713, Doha, Qatar; 2 ConocoPhillips Global Water Sustainability Center, Qatar Science, and Technology Park, P. O. Box 24750, Doha, Qatar; 3 Materials Science and Technology Graduate Program, College of Arts and Sciences, Qatar University, P. O. Box 2713, Doha, Qatar

**Keywords:** Oil removal, Emulsions, Melamine foams, Absorption capacity, Superhydrophobicity, Superoleophilicity

## Abstract

This study deals with the efficient, low-cost, and scalable treatment of oily polluted waters including colloidal emulsions, oil-in-water mixtures, and free oil removal using melamine foams (MFs) modified by ferric chloride (FeCl_3_). Modified foams have superhydrophobic character due to the coordination of Fe^3+^ with free electron pairs on nitrogen and oxygen atoms within the melamine structure. The water contact angles (WCA) were 146° ± 2°, 148° ± 4°, 153° ± 2°, and 150° ± 4° for foams modified by the solutions with concentrations of 0.001 M, 0.005 M, 0.01 M, and 0.02 M, respectively. This modification enables the efficient treatment of various oil/water systems, including oil/water colloidal emulsions (99 vol% of the droplets have dimensions below 500 nm), oil-in-water mixtures up to 40 weight % of the oil component, and “free” oil removal as it was demonstrated in this study for the first time. The emulsions containing 100 ppm diesel oil (DO) were separated with 91.4% efficiency, and the mixtures containing 20 and 40 weight % DO were separated with 99.9% efficiency. Modified foams also quickly remove free DO from the water surface, absorbing 95 g/g DO, whereas water sorption was negligible. The separation of colloidal oil in water emulsions represents the key finding of this study as it indicates the applicability of the treated MFs for the treatment of emulsified industrial wastewater. The demulsification mechanism is based on multiple diffusion processes running at different time scales, including diffusion of the emulsion into the foam and diffusion of oil droplets within the foam, combined with parallel adsorption of oil droplets onto the solid skeleton of the foam. A multiplied usage of these foams for all these niche operations was also proven. The application of our current study with previous studies on modified MFs and polyurethane for water oil separation utilization is summarized in Table S1 ESI.

**Graphical Abstract:**

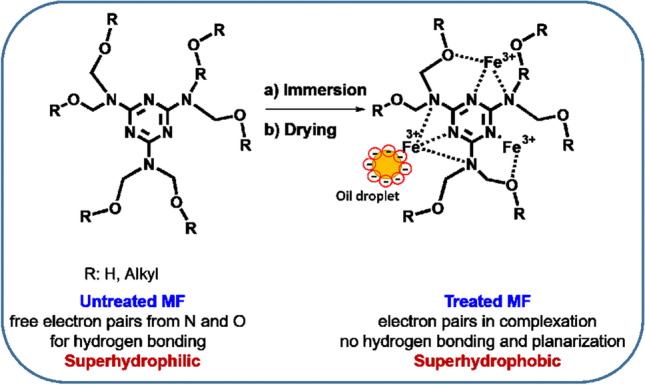

**Supplementary Information:**

The online version contains supplementary material available at 10.1007/s11356-023-29055-x.

## Introduction

Oil and grease (O&G) are organic compounds (saturated and unsaturated hydrocarbons, fatty acids, waxes, etc.), which, if spread in water, have a serious impact on the biosphere, aquatic life, and, from a practical point of view, on the enhanced costs associated with water purification for common uses (Ding et al. 2018a; Pintor et al. 2016; Aguilera et al. 2010). Most of these compounds have very little solubility in water; however, the intensive use of surfactants in oil refining, the petrochemical industry, food processing, cosmetics, and households (Neff et al. 2011; Al-Ghouti et al. 2019) leads to the dispersion of oily components within the water at various sizes and concentration scales. The removal of O&G from water depends on the composition and morphology of water/oil systems as well as on the magnitude of treated volumes and targeted purity. The morphology of oil/water systems primarily determines the treatment strategy. According to the most commonly cited classification introduced by Patterson and Patterson (1985) based on the droplet size, the oil in water can occur as (i) free oil mostly floated on the water surface (droplets’ diameter range > 15 μm, (ii) dispersed oil 20–150 μm, (iii) emulsified oil (< 20 μm), and (iv) soluble or dissolved oil (< 5 μm). The removal of the free oil is called the primary treatment, and the main methods used are based on gravity separation resulting in different densities of water and organic pollutants. Secondary treatment is focused on the removal of dispersed organic contaminants using various physical (mechanical), electrical, chemical, and biological methods (Pintor et al. 2016). Finally, tertiary treatment is employed as the last step of water purification, and the target effluent is < 10 mg/L (Dickhout et al. 2017). Current tertiary filtration technology mostly includes membrane filtration (Dickhout et al. 2017; Adham et al. 2018; Tanudjaja et al. 2019), synthetic resins (Albatrni et al. 2019), and walnut shell filtration (Rahman 1992; Yin et al. 2020). The difficulty of oil separation increases with a decrease in droplet size and with an increase in the stability of mixtures due to various surfactants commonly used in industry (Neff et al. 2011; Al-Ghouti et al. 2019). Many existing water sources, particularly those produced by the petrochemical industry, contain oil droplets or insoluble organic components with sizes around or below 1–2 μm and less, and the treatment of such systems represents the most challenging problem (Al-Ghouti et al. 2019; Patterson and Patterson 1985; Dickhout et al. 2017).

Adsorption is considered a very efficient and versatile method for the removal of contaminants from water (Dąbrowski 2001). It enables the removal of droplets with colloidal dimensions, which cannot be separated by common filtration techniques as deep as filtration using a walnut shell medium (Rahman 1992; Yin et al. 2020), e.g., special types of sorbents represent foams, especially polymeric foams such as cellulose (Abu-Thabit et al. 2022), polydimethylsiloxane (Zhang et al. 2021), melamine (Hailan et al. 2021), and polyurethane foams (Hailan et al. 2021; Pinto et al. 2018; Vásquez et al. 2019; Oliveira et al. 2021).

Melamine foams (MFs) are particularly suitable for various routes of oil/water separations due to their high porosity; chemical, thermal, and mechanical stability; easy availability; and low cost (Hailan et al. 2021; Zhang et al. 2020; Ding et al. 2018b). Moreover, many protocols have been reported for modifying their wettability to attain (oleo)hydrophilic or (oleo)hydrophobic surfaces (Dashairya et al. 2020). These approaches involve carbonization (Chen et al. 2013), attachment of graphene (Han et al. 2021a) and silica nanoparticles (Li et al. 2015), coating by polydimethylsiloxane (Ong et al. 2019), silanization (Wang et al. 2018), and others. Furthermore, MFs possess very high elasticity and mechanical integrity, enabling them to be reused for thousands of cycles (Hailan et al. 2021). Finally, the low-cost production of suitable sorbents is another crucial point for industrial applications due to the large volumes of treated water (Han et al. 2021b, 2022; Li et al. 2022). A very efficient and simple and low-cost procedure for MF treatment has been published very recently by Ding et al. (2018a). In this study, they described a facile fabrication method for the treatment of MF through their short immersion time into water solutions of various transition metal salts, such as FeCl_3_, Fe(NO_3_)_3_, Ni(NO_3_)_2_, Zn(NO_3_)_2_, and Co(NO_3_)_2_. The coordination bond between transition metals and free electron pairs on nitrogen atoms within repeating 2,4,5-triamino-s-triazine structural units induces a transition between hydrophilic and hydrophobic states (Ding et al. 2018a). This inspires us for a comprehensive study on affordable and sustainable Fe-based MFs for the treatment of different water–oil, water-diesel oil, water–oil separation, and importantly, challenging treatment with oil droplets with sub-micron droplet size. The previously published works investigated a transition of melamine foam from hydrophilicity to hydrophobicity that was induced by the different ions. This treatment resulted in high oil sorption and in suppression of water sorption regardless of the type of ions, just considering the ions’ concentration. The presented work is the first study focusing not only on the sorption of pure oil (meaning various organic non-polar liquids) but also on the separation of o/w mixtures including both high oil content mixtures and emulsified mixtures having very low oil content. It was revealed that Fe^3+^ cations embedded in foam structure generate an electrostatic field, which contributes to the adsorption of negatively charged species. Such material feature enables the demulsification of stable emulsions with negative zeta potential, even at a colloidal scale.

## Materials and methods

### Sorbent media

Commercial melamine foams (LTWHOME, Carrefour, Qatar) were washed in distilled water, dried, and cut into 1.5 × 1.5 × 1.5 cm cubes. Solutions of ferric chloride (FeCl_3_, 97%, Research-Lab Fine Chem Industries) at four different concentrations (0.001 M, 0.005 M, 0.01 M, and 0.02 M) were prepared. The modification of MFs was performed through immersion into ferric chloride solutions at room temperature for 30 min under continual shaking. Then, solutions were squeezed out of the foams, and the foams were washed with distilled water and dried in a vacuum oven at 60 °C. Diesel oil (WOQOD, Qatar) was used for the preparation of emulsions and mixtures by mixing with distilled water.

*Preparation of* oil in water emulsions and mixtures.

The emulsions were prepared by mixing aliquot amounts of diesel oil (DO) and distilled water through sonication by a probe sonicator for 15 min at room temperature at 40% amplitude using an ultrasonic sonicator (HIELSCHER UP400S, Berlin, Germany) with a 22-mm titanium probe used as the homogenizer.

The mixtures were prepared by mixing DO and distilled water in ratios of 20/80 and 40/40 weight % by vigorous mechanical shaking for 5 min.

All tests using emulsions and mixtures were performed immediately after their preparation.

DO was used in this study because it represents a reasonable, easily available model of the hydrocarbon fractions occurring in refinery wastewaters or produced waters instead of various grades of crude oil. It is composed of approximately 75% saturated alkanes (linear alkanes and monocyclic-alkanes) and approximately 25% aromatic hydrocarbons (naphthalenes and alkyl benzenes) (Diraki et al. 2019; Gulyas and Reich 1995).

### Batch sorption test

The sorption experiments for both emulsions and mixtures were performed in 50-ml volume glass Falcon test tubes. The size of the tested foams was arbitrarily chosen to be 1.5 × 1.5 × 1.5 cm. The number of testing foams varied from 1 to 7 pieces. Testing foams were inserted into tubes, and tubes were filled with testing emulsions or mixtures. Sorption experiments were performed over the selected periods at 22 ± 1 °C. The tubes were shaken by a mechanical shaker to suppress the formation of concentration gradients within emulsions and mixtures for the duration of the experiment. At the end of each test, foams were removed from a tube, and the liquid was squeezed out of foams one by one using a syringe to control constant squeezing conditions. The oil content in the permeate and in the liquid, which was squeezed out of the foam (squeezed permeate), was determined by the measurement of total organic carbon (TOC) in the samples. The TOC values of the testing emulsions (batch emulsions) were always remeasured at the beginning of each sorption experiment, and these values were used for further calculations. Each sorption experiment was repeated at least three times.

The reuse of foams was tested by multiple sorption/desorption experiments. The foams employed for multiple testing were used immediately after finishing the previous cycle, without any additional cleaning or treatment. This procedure was repeated 10 times.

Sorption of water and free oil was performed by placing foams on the surface of water or oil, and the increase in the mass of foams was determined in selected intervals.

### Surface morphology analysis and elemental analysis

The surface topography of unmodified and modified MFs was analyzed using an optical surface metrology confocal system profilometer (Leica DCM8; Leica Microsystems, Germany). The optical system was used for high-accuracy surface profiling to optimize the PLA fiber mat formation. The 3D micrographs of 876.55 × 659.83 μm^2^ were recorded using an EPI 20X-L objective lens. In addition, surface roughness was evaluated according to an arithmetic mean height value (Sa).

The morphology and composition of unmodified and modified MFs were examined by a field emission scanning electron microscope (FE-SEM, Nova Nano SEM 650) equipped with energy dispersive X-ray spectroscopy (EDS) by secondary electron images with 3 kV and different magnifications. All specimens were sputter coated with 2 nm gold before SEM.

Fourier-transform infrared spectroscopy (FTIR) (Spectrum 400, PerkinElmer, USA) was employed to investigate the influence of the coordination of FeCl_3_ on melamine structure. FTIR spectra were recorded in the wavenumber range of 500–4000 cm^−1^.

### Surface wettability characterization

The surface wettability of foams by water and DO was characterized by contact angle measurements using System OCA 35 (Dataphysics, Germany). The measurements were performed under air, and DO was also measured underwater.

### Characterization of emulsions by dynamic light scattering (DLS) and zeta potential

Zetasizer Lab (Malvern Panalytical, UK) was used for the determination of both droplet sizes by dynamic light scattering (DLS) and the zeta potential of emulsions. The DLS measurements were performed in glass cuvette DTS1070 using 12 μl of emulsions, and the zeta potential was measured in ZS90 cuvettes using 2 μl of emulsion. Each measurement was repeated three times.

### Total organic carbon analysis (TOC)

TOC analysis was performed using a Formacs TOC/TN analyzer (Analytikjena, Germany). The samples were injected into a high-temperature combustion furnace where organic carbon (OC) was converted to carbon dioxide at 850 °C by catalytic oxidation (Pt catalyst). The formed CO_2_ was then dispersed into the carrier gas, and the concentration was measured by using a nondispersive infrared detector (NDIR).

### The morphology of oil in water mixture characterization

The morphology of oil in water mixtures was analyzed using an optical surface metrology confocal system profilometer (Leica DCM8; Leica Microsystems, Germany). The optical system was used for the high-accuracy analysis of the oil distribution and morphology in water before and after the filtration process. Images of 350.62 μm × 263.93 μm and 876.55 μm × 659.83 μm were captured using EPI 50X 0.8-L and EPI 20X-L objective lenses, respectively.

## Results and discussion

### Characterization of unmodified and modified foams

**Fig. 1:**
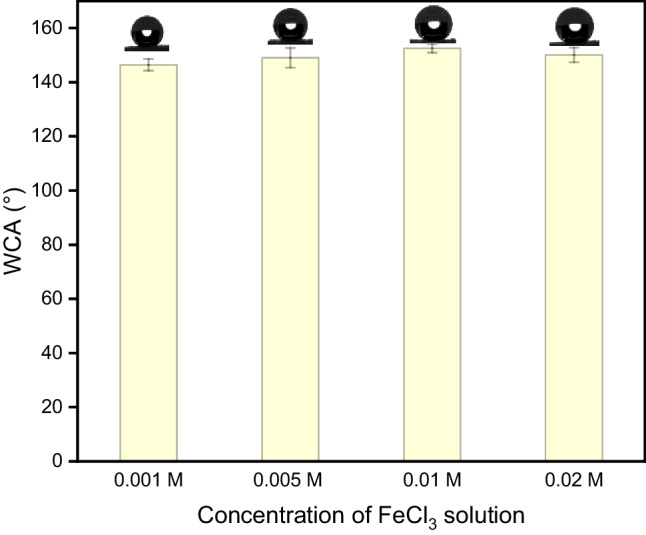
Water contact angle of modified melamine foams (MFs) with the corresponding image above the value at a concentration of FeCl_3_ solution

MFs are inherently supehydrophilic and superoleophilic, and therefore, for oil/water separation applications, they have to be modified to obtain hydrophobicity and maintain oleophilic character. Modification of MFs was carried out with Fe^3+^ ions since it is affordable, naturally abundant as part of biologically active compounds, and environmentally benign (Fredrick et al. 2010; Qin et al. 2022). The modification was performed by immersion in 4 different concentrations of FeCl_3_ of 0.001 M, 0.005 M, 0.01 M, and 0.02 M FeCl_3._ All modified samples showed superoleophilic behavior with CA of about 0° for DO in air and underwater. Modified foams possess a highly or superhydrophobic character. The water contact angles (WCA) were 146° ± 2°, 148° ± 4°, 153° ± 2°, and 150° ± 4° for foams modified by the solutions with concentrations of 0.001 M, 0.005 M, 0.01 M, and 0.02 M, respectively (Fig. 1). It should be noted that the WCA reported by Ding et al. (2018a) was much lower ranging from 121 to 127° for the same concentrations. Differences can be attributed to different micro/nanostructured surfaces of melamine foam, and therefore, further morphology and chemical analysis were carried out. MFs modified with a concentration of 0.01 M FeCl_3_ was chosen for further studies due to the highest water contact angle.

**Fig. 2:**
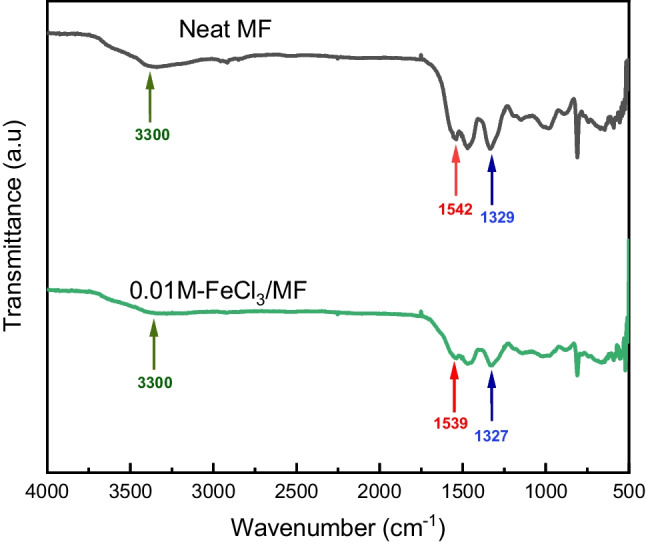
FTIR spectra of the unmodified and modified MFs

The influence of the coordination of FeCl_3_ on melamine structure was investigated by FTIR (Fig. 2). The unmodified ME sample showed a large absorption band at 3300 cm^−1^ attributed to hydrogen bonding of N–H or OH group to free electron pair to nitrogen heteroatom in ME structure, while this band was dramatically reduced in the modified sample. Moreover, the unmodified sample showed bands at 1542 cm^−1^ and 1329 cm^−1^ corresponding to the stretching vibration C = N and C–N bands from the melamine triazine ring, respectively (Stuart 2004). However, these bands shifted in the modified sample to 1539 and 1327 cm^−1^. These shifts indicated the decreasing strength of such bonds due to the reduction of electronic density on N and involvement in the coordination of the free electron pair of the nitrogen atom with the Fe^3+^ metal ion.

**Fig. 3:**
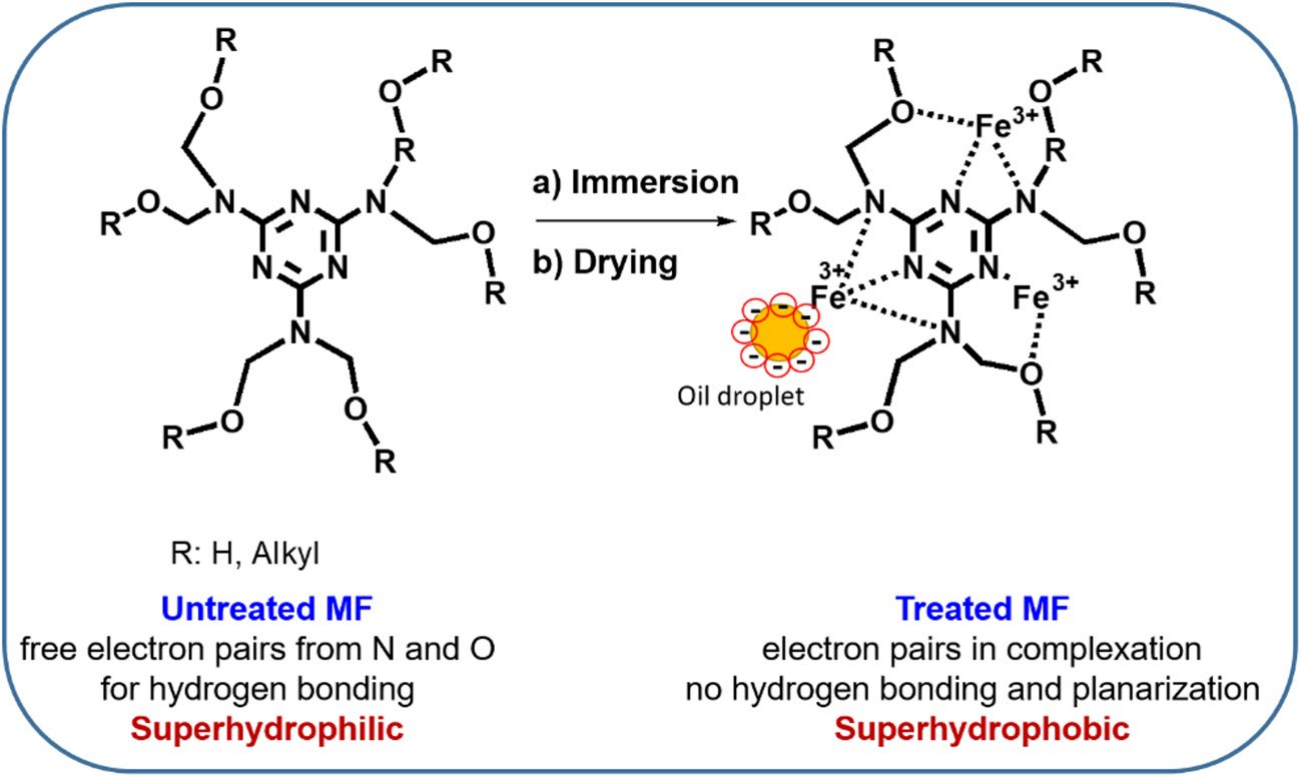
The schematic presentation of the mechanism of coordination of melamine structural unit and ferric chloride and charged oil droplet-MF interaction

The transition from hydrophilicity to hydrophobicity has a crucial influence on the functionality of MF foams for applications discussed in this paper. Ferric (III) chloride strongly coordinates with free electron pairs on nitrogen and oxygen atoms within the melamine structure. The reduction of electron density on nitrogen due to coordination with Fe^3+^ cations inhibits the further activity of nitrogen and oxygen as electron pair donors. Moreover, the complexation of ferric (III) ions might lead to enhanced planarity of melamine units (Ding et al. 2018a). The proposed mechanism of coordination of the melamine structural unit and ferric chloride is depicted in Fig. 3. This coordination subsequently prevents nitrogen from being active in hydrogen bonding and increases the aromatic character of the melamine-based structure, leading to a dramatic decrease in wettability. Additionally, charged positive Fe^3+^ ions in the structure initiate disruption of negatively charged emulsion leading to the coalescence of oily based droplets as will be discussed below.

**Fig. 4:**
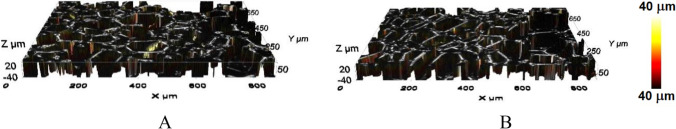
Profilometer micrographs of unmodified (**A**) and modified (**B**) MFs

Surface topography of unmodified and modified MFs in 876.55 × 659.83 μm^2^ surface area was obtained by profilometry technique, and 3D micrographs are shown in Fig. 4. The specific porous structures were observed in both unmodified and modified MFs. The modification of MF by FeCl_3_ led to a rougher outer layer of fibers while large pores remained intact. The surface roughness of unmodified MFs, represented by Sa value (arithmetical mean height), was 135.35 µm for unmodified MFs. An additional modification of MFs by FeCl_3_ showed an increase in surface roughness, while Sa was 169.35 µm.

**Fig. 5:**
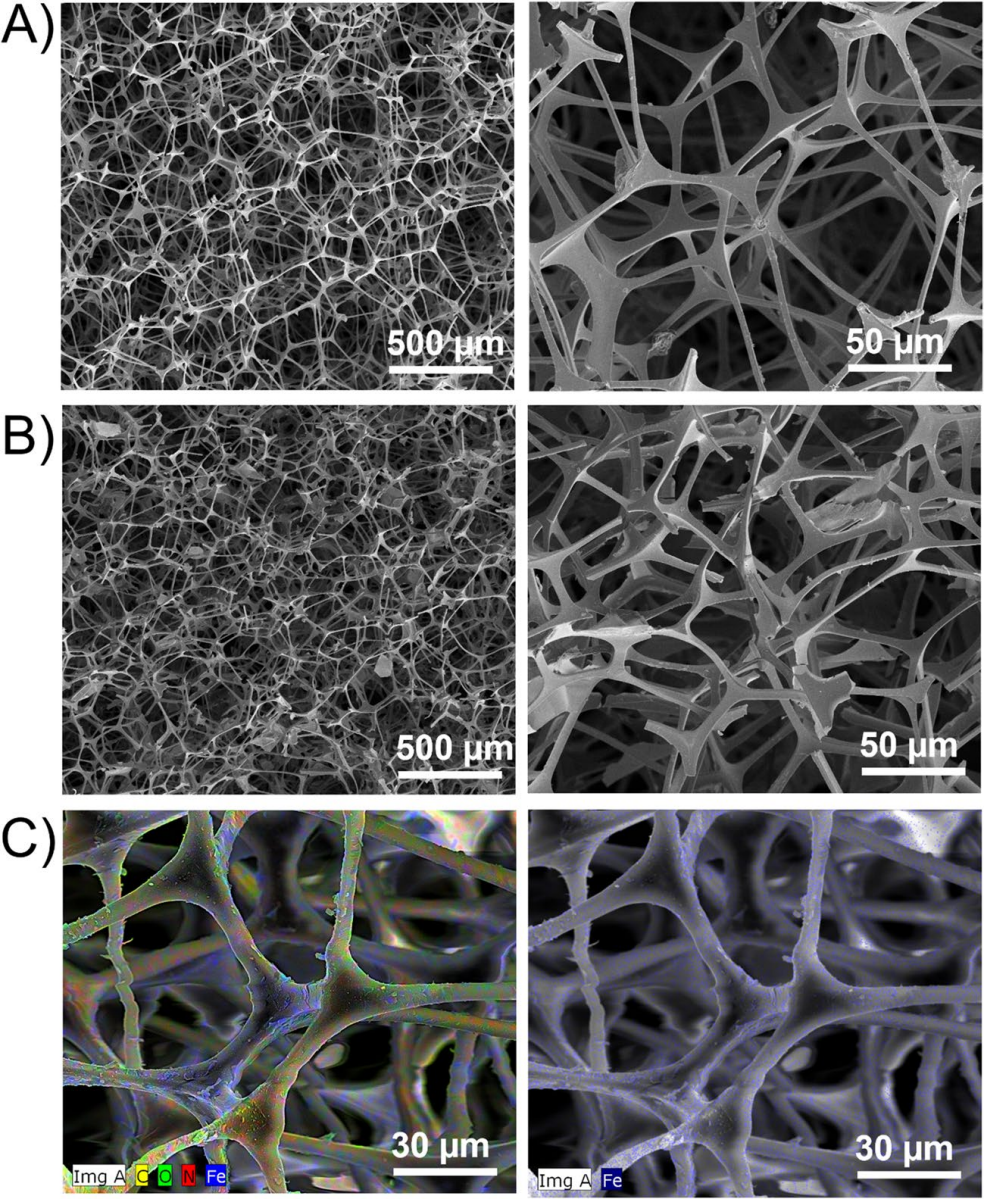
SEM micrographs of unmodified (**A**) and modified MF (**B**), EDS mapping of modified MF (**C**)

The SEM micrographs (Fig. 5A, B) at lower magnification did not show significant changes in MF morphology after modification. The sample retained its open-cell foamy structure and its smooth fibrous network. At higher magnification, a low amount of small particles was observed on the edges of fibers in treated foams, attributed to aggregated architectures after Fe^3+^ coordination to foam structure that is in agreement with the profilometry study. EDS analysis was performed for the cross-section of the modified MF. Figure 5C shows the distribution of Fe on the internal surface of the foam. The distribution was uniformly distributed in fibrous structure and homogenous. The weight content of the detected atoms is as follows: 32.8 ± 1.4 wt.% of C, 41.6 ± 1.7 wt.% of N, 13.6 ± 1.9 wt.% for O, 5.4 ± 0.1 wt.% for Fe, and 5.1 ± 0.1 wt.% for Cl. Carbon and nitrogen belong to the melamine structure, and Fe and Cl come from ferric chloride. Oxygen is associated with adsorbed moisture.

The density of unmodified and modified MFs was determined from their mass and volume before and after treatment. Foam with an original size of 6.016 × 9.097 × 2.951 cm^3^ was used. The density of the pristine MFs was (8.07 ± 0.15) × 10^−3^ g.cm^−3^, and the density of the treated MFs was (10.26 ± 0.19) × 10^−3^ g.cm^−3^. The porosity of foams (*ϕ*_p_) was estimated from Eq. (1):

1 $${\phi }_{p}\left(\%\right)=\left(1-\frac{{\rho }_{f}}{{\varrho }_{m}}\right)\times 100\%$$

where *ρ*_m_ = 1.51 g.cm^−3^ was taken as the density of the solid melamine resin (Ding et al. 2018a). The porosity of unmodified MFs was found to be 99.47 vol%, and the porosity of modified MFs was 99.32 vol.%.

### Characterization of DO/water emulsions

The oil-in-water emulsions are prepared using surfactants, which reduce the free energy at the oil/water interface, enabling fine dispersion of oil droplets within the water medium and maintaining the kinetic stability of emulsions. Another possibility is emulsification without any surfactants using ultrasonication of mixtures at high frequencies, the method employed in this study. This process generates various active oxygen species (OH radicals, OH^−^, and hydrogen peroxides) that interact with the oil droplets’ surface and alter the electric charge at the oil/water interface (Takahashi and Sakamoto 2017). This charge is negative, as can be confirmed by the measurement of the zeta potential. The zeta potential of the emulsions investigated in this study was approximately − 34 mV for emulsions containing 100 ppm DO and approximately − 37 mV for emulsions containing 1000 ppm DO, indicating moderate kinetic stability of both emulsions. The 100 ppm emulsion was selected as a model emulsion for the tertiary treatment of produced water (Dardor et al. 2021), and the 1000 ppm emulsion was used as the “masterbatch” for the preparation of emulsions with the required concentrations because it was found during this study that the accuracy of the emulsion concentration can be better insured by dilution of a “masterbatch” than through the direct preparation of emulsions.

**Fig. 6:**
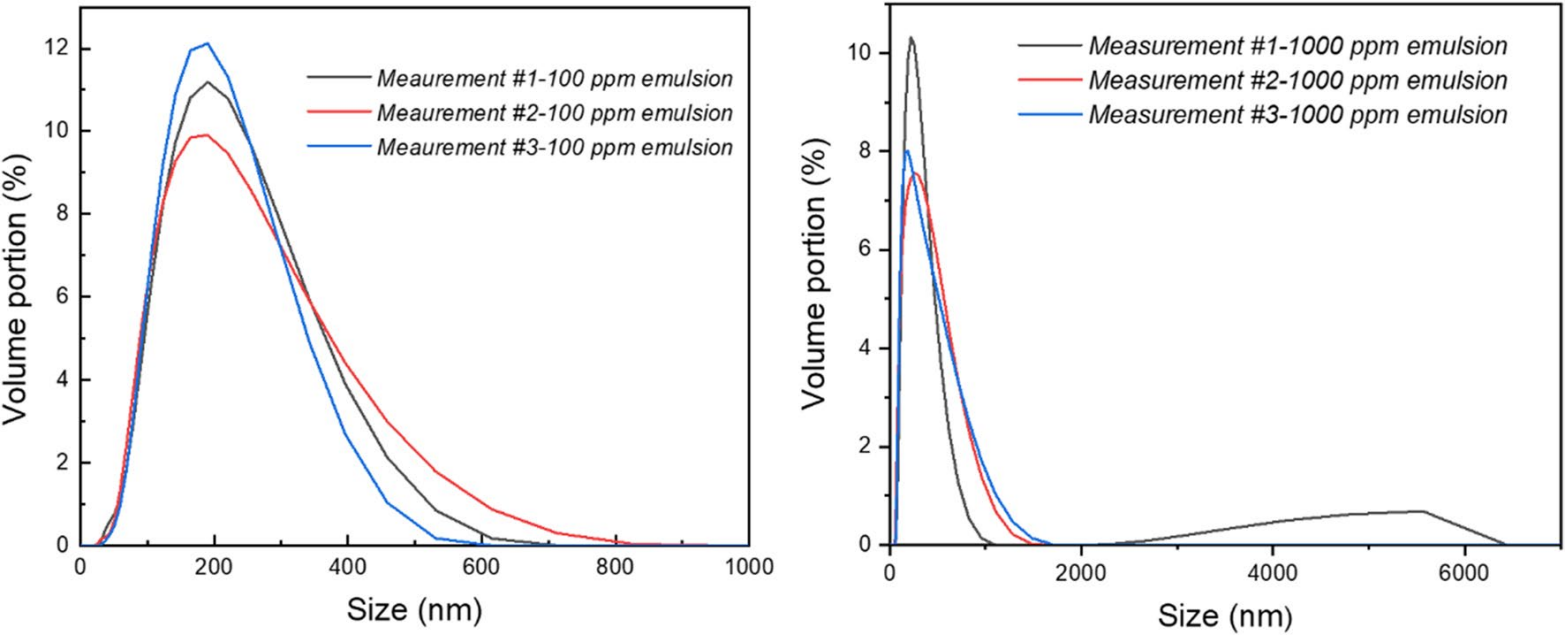
The differential distribution curves of oil droplet size. Left, 100 ppm emulsion; right, 1000 ppm emulsion

The droplet size distribution was characterized by the dynamic light scattering method. The differential distribution curves for 100 ppm and 1000 ppm emulsions are shown in Fig. 6. For illustration, only three representative measurements for each emulsion were selected, demonstrating the reproducibility of emulsion preparation. The distribution curves of the emulsions slightly differ, as shown in Fig. 6 and the data in Table 1. For the 100 ppm emulsion, 99 vol% of the droplets have dimensions below 500 nm, and 100% of the droplets have dimensions below 1000 nm, which confirms the colloidal character of this emulsion. The distribution curve of the 1000 ppm emulsion also has 99 vol.% of droplets within the colloidal region; however, there is a very small fraction (approximately 1%) of droplets with sizes in the range from 2000 to 6000 nm.

**Table 1:** Distribution of droplet sizes and zeta potentials of 100 ppm and 1000 ppm emulsions

Emulsion	Size at peak (nm)	Size < 100 nm (%)	Size < 200 nm (%)	Size < 500 nm (%)	Size < 1000 nm (%)	Size < 2000 nm (%)	*Z*-potential (mV)
100 ppm	187 (4)	13 (1)	60 (3)	99 (1)	100	100	− 34.0 ± 0.6
1000 ppm	178 (8)	10 (2)	61 (4)	97.4 (0.7)	99 (1)	99 (1)	− 37.0 ± 0.5

The data in Table 1 refer to selected values taken from the differential particle size distribution curve expressing the percentage of droplets from the total oil volume content that is within a specified size range.

The concentrations of 100 and 1000 ppm are analytical concentrations of DO used for the preparation of emulsions. In the following text, the exact concentrations of carbon (TOC values) in emulsions determined by a TOC analyzer for each investigated are used instead of analytical concentrations of DO. The TOC values differ from the analytical concentrations of DO. This is because the content of carbon in DO is approximately 80 ± 3 wt.% as determined by elemental analysis, and second, there are always some losses of oil content in emulsions as a consequence of sample preparation and manipulation. For this reason, it is much more precise to determine the exact TOC value before any experiment. For example, emulsions having an analytical concentration of DO equal to 100 ppm as prepared show TOC values of approximately 73 ± 4 ppm.

### Characterization of DO/water mixtures

**Fig. 7:**
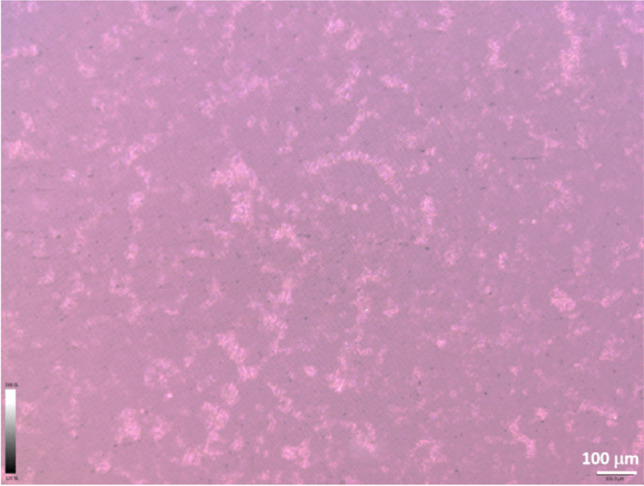
The morphology of oil-in-water mixtures (o/w = 20/80 w/w). The lighter the color-oil phase is, the darker the color-water phase

The morphology of oil-in-water mixtures analyzed by the optical surface metrology confocal system profilometer is shown in Fig. 7. The figure shows that oil/water mixtures form the co-continual structure in which the oil component forms both discrete and percolating structures dispersed within the major, continual water phase. These mixtures tend to be phase separated over time, leading to the formation of free oil on the surface; therefore, sorption tests were performed immediately after mixture preparation.

### Separation of DO/water emulsions

The separation of emulsified o/w mixtures is the most challenging application of modified MFs, particularly the purification of low-concentrated colloidal emulsions having droplet sizes below 500 nm. In this case, Fe^3+^ cations not only induce hydrophobicity of foam but they also cause oils’ droplet adsorption on the internal surface of MFs. In the first stage, the emulsion slowly penetrates a foam, and then negatively charged oil droplets are electrostatically attracted by Fe^3+^ cations. It results in a decrease in oil concentration in the absorbed liquid. Consequently, it leads to the formation of the concentration gradient between the emulsion within a foam and the emulsion in the surroundings resulting in the diffusion of the oily droplet into the foam. Consequently, it leads to the formation of the concentration gradient between the emulsion within a foam and the emulsion in the surroundings resulting in the diffusion of the oily droplet into the foam and decreasing oil content in the filtrate until reaching the equilibrium. This process is investigated and described in the next paragraphs.

#### Diffusion of water and emulsions into the foams

**Fig. 8:**
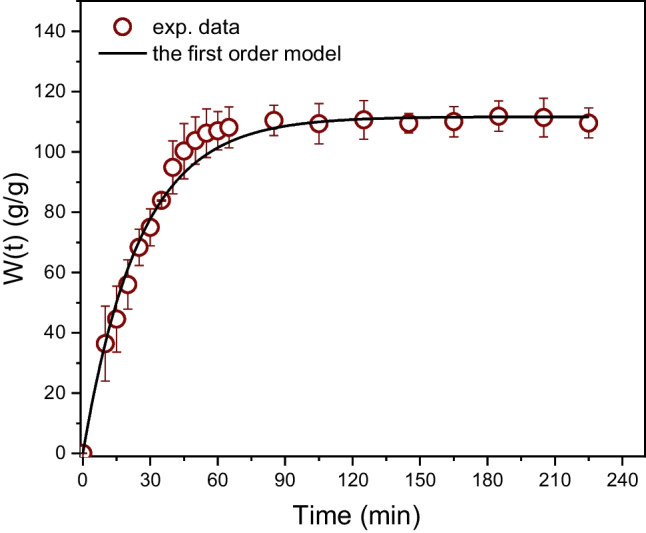
Time dependence of water/emulsion sorption into the treated MFs (1.5 × 1.5 × 1.5) cm. The solid line, Eq. (4)

The modification of MF leads to the formation of (super)hydrophobic surfaces, as previously discussed. Superhydrophobicity is a consequence of the combined effects of chemistry ensuring the low surface energy of materials and surface topology associated with an appropriate roughness and can be explained and described by the Wenzel model (Wenzel 1936) or the Cassie-Baxter model (Cassie and Baxter 1944). However, in some cases, hydrophobicity may not necessarily suppress the diffusion of water (or emulsions) into the foams, particularly if they are immersed within the water, the system is shaken or stirred, and the time of water exposure is sufficiently long. In this case, pores or voids on the material surface, which are initially filled by air, can be refilled by water, and then the penetration of liquid runs into the foams. However, water diffusion is significantly decelerated due to the chemical modification of foam in the whole volume. In other words (in our case), the hydrophobicity induced by Fe^3+^ cations still plays a role. This phenomenon is crucial for the separation of oil emulsions by foamy structures. In this study, the concentration of the oil component dispersed in water ranges from tens to hundreds of ppm, so the major phase is water, which acts as the carrier of oil droplets transporting them into the internal structure of the foam. In other words, oil droplets themselves cannot diffuse into a porous foam filled with air only. Figure 8 shows the time dependence of the amount of absorbed water within a treated foam (1.5 × 1.5 × 1.5 cm). The dependences of emulsion absorption investigated in this study were the same, within the range of the experimental error.

Figure 8 shows that the foam is fully saturated by liquid approximately 60 min after immersion. The time of foam saturation depends on its size and shape, as is known from a general theory of diffusion (Wenzel 1936; Cassie and Baxter 1944). The size and shape of the testing foam were arbitrarily chosen, and all the foams used in this study had the same shape and size in all experiments.

The experimental data can be perfectly fitted over the whole time scale by the first-order kinetic model given by Eq. (4). *W* [*g*/*g*] is the mass of the absorbed water per mass of the sorbent (foam) and *k*_1_ [min^−1^] is the rate constant.

This model also enables quantitative characterization of an equilibrium state (*W*_e_)—Eq. (2)—and the rate of sorption at any selected time (*v*(*t*))—Eq. (3).

2 $$\begin{array}{c}W\left(t\right)={W}_{e}\left(1-\mathrm{exp}\left(-{k}_{1}t\right)\right)\\ {W}_{e}=\underset{t\to \infty }{\mathrm{lim}}W(t)\end{array}$$

3 $$v\left(t\right)=\frac{dW(t)}{dt}={W}_{e}{k}_{1}exp(-{k}_{1}t)$$

The parameters *W*_e_, *k*_1_, and *v*_0_ are summarized in Table 2.

**Table 2:** The parameters of the first-order model and the power law model

Model		Parameters		
$W\left(\mathrm{t}\right)=A{t}^{n}$	*A* [min^−*n*^]	*n* [ −]	*k*_D_^*^ [min^−*n*^]	*R*^2^
	8 (2)	0.67 (0.07)	0.071	0.99832
$W\left(t\right)={W}_{\mathrm{e}}\left(1-\mathrm{exp}\left(-kt\right)\right)$	*W*_e_ [g/g]	*k*_1_ [min^−1^]	*v*_0_^**^ [(g/g).min^−1^]	*R*^2^
	112 (2)	0.039 (0.001)	9.0	0.99996

^*^ k_D_ was calculated from *A* using *W*_e_ = 112 g/g

^*^ *v*_0_ was calculated from Eq. (5) for* t* = 0

On the other hand, from a physical point of view, the penetration of liquids into foam is a diffusional process. The most common model for a description of such processes is the generalized non-Fickian diffusional model, given by Eq. (4), which was originally introduced by Ritger and Peppas 1987a; Ritger and Peppas 1987b) for the interpretation of a non-Fickian release of drugs from moderately swelling polymeric systems.

4 $$\frac{{M}_{t}}{{M}_{\infty }}=k{t}^{n}$$

**Fig. 9:**
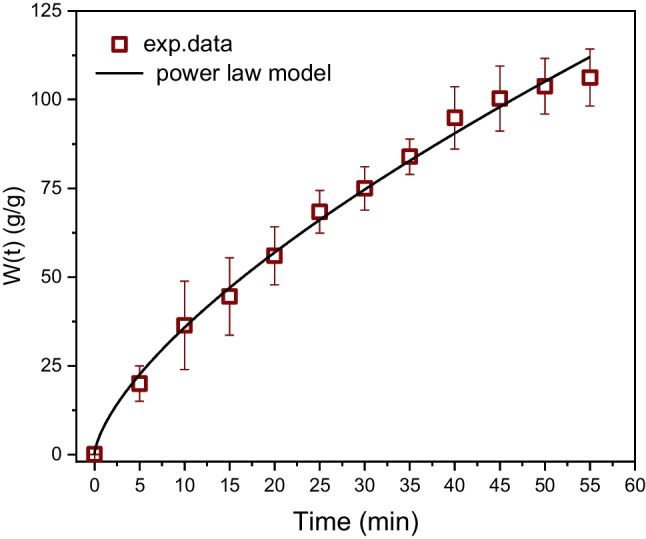
Time dependence of absorbed water within a treated foam (1.5 × 1.5 × 1.5 cm). The solid line, Eq. (7). *W* is the mass of the absorbed water per mass of the sorbent

In the original papers, *M*_t_ and *M*_∞_ are mass concentrations of a released species at time *t*, and at the time approaching infinity, *k* is a constant involving characteristic of the network (medium) and the species, and *n* is a diffusional exponent. This model is broadly applied for a description of processes controlled by diffusion (Fig. 9). However, the fitting of data by Eq. (4) is restricted only for times when the sorption values do not reach a plateau (from zero to approximately 60 min). Equation (4) was applied to fit the experimental data on this time scale; however, for practical reasons, Eq. (4) was rewritten into Eq. (5), which better characterizes a portion of the absorbed liquid within a foam in proportion to the weight of a pristine foam.

5 $$W\left(t\right)=\frac{m(t)}{{m}_{0,f}}=A{t}^{n}$$

where *m*_0,f_ [g] is the initial weight of the foam, *m*(*t*) is the weight of the foam at time *t*, and *A* = *km*_∞_/*m*_0,f_.

The fitting of experimental data by Eq. (5) gives the parameters *A* = 8 ± 2 [min^−*n*^], and *n* = 0.67 ± 0.07). In general, if *n* = 1/2, the model characterizes Fickian diffusion, and *n* > 0.5 indicates so-called anomalous diffusion, which, unlike Fickian diffusion, runs in heterogeneous systems consisting of diffusional boundaries, such as pores, swollen and dry regions, regions with different physical states (glassy, rubbery), etc. (Ritger and Peppas 1987a, b).

#### The influence of the dosage

It is generally known that the separation efficiency of sorbents depends on the ratio of the dosage of sorbent and the volume of treated emulsions (Pintor et al. 2016). The amount of sorbent is usually (for practical reasons) expressed in mass units, but it may also be expressed in volumetric units. Strictly speaking, the key parameter that influences a sorption process is neither the mass nor volume of the sorbent, but it is the surface area, which is accessible for sorption. In the case of smooth objects, the surface area is directly related to the volume; in the case of porous materials, this relation is less unambiguous, as a surface area has fractal dimensions. Therefore, the mass is practically the simplest parameter to express the relative sorption parameters. On the other hand, the mass of sorbents is a less relevant parameter in the case of bulky foams located in a finite volume (a separation column or vessel, e.g.), where a volume of absorbents dictates real applicability. In the following text, the ratio between the volume of used foams and the volume of treated emulsions is considered, and the volume of foams is arbitrarily expressed by the number of used foams. The volume and size of each foam are always constant (1.5 × 1.5 × 1.5 cm) with a mass of 0.027 ± 0.001 g and the volume of the treated emulsion is equal to the volume of the testing tube (50 mL). The dimensions of foams were selected arbitrarily, just for a practical reason to allow for simple manipulation. However, it is clear that, unlike equilibrium experiments, the kinetics of sorption is influenced by the shape and size of foams because they are controlled by diffusion; therefore, kinetic experiments must be performed individually for any specific geometry.

In the first stage, the number of cubes for further testing was optimized. For this reason, multiple experiments were performed to test DO removal from 100 ppm emulsion at a constant time of 3 h using 1-, 3-, 5-, and 7-modified foams. As expected, it was found that the number of foams enhanced sorption efficiency up to some limit. According to the sorption experiment data, the separation efficiencies of foams 1, 3, 5, and 7 were 47%, 66%, 82%, and 84%, respectively, as a consequence of increasing the contact area. Using more foams did not lead to the improvement of sorption efficiency, and therefore, seven foams were used for all further sorption experiments. For comparison, seven untreated MFs as sorbents were tested under the same conditions, and the separation efficiency was approximately 9%.

#### The influence of the initial concentration of DO in emulsions

**Fig. 10:**
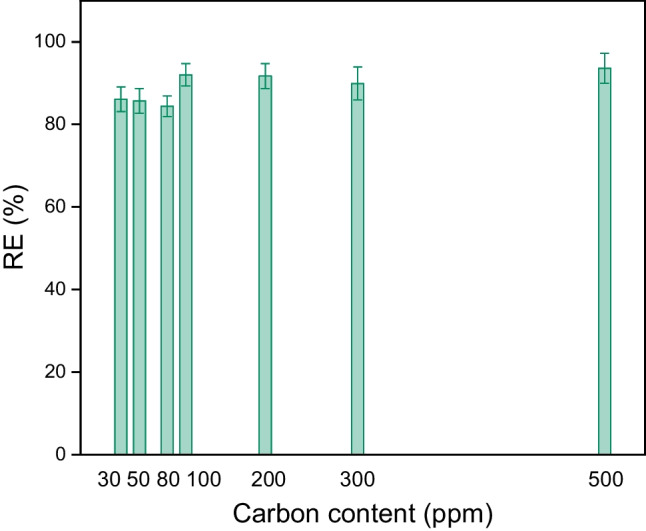
The dependence of removal efficiency (RE) on the initial concentration of DO (expressed in the form of carbon content)

The absorption experiment was performed using seven foams immersed in 50 mL of emulsion with different DO contents at 22 °C for 24 h. The concentrations chosen for these experiments are related to the concentration range of oil impurities in the water produced, which is characteristic of the tertiary and secondary treatments of emulsified water, and it ranges from 30 to 550 ppm of carbon in the emulsion. Figure 10 shows that the removal efficiency of foams slightly increases with an increase in the concentration of stock solutions from approximately 86 to 94%.

The removal efficiency (RE) is defined by Eq. (6):

6 $$RE [\%]=\frac{{c}_{0}-{c}_{e}}{{c}_{0}}\times 100\%$$

where *c*_0_ [mg/L] is the initial concentration of oil in the emulsion and *c*_e_ [mg/L] is the concentration of oil in the emulsion at equilibrium.

This is a logical tendency; however, these data do not enable the construction of any reasonable adsorption isotherm. There can be a few reasons for this. First, it is difficult to determine the exact amount of adsorbed oil on the skeleton surface inside the foam, and second, the multistep sorption process may not fulfill the conditions at which various adsorption isotherms were derived.

#### Kinetics of DO sorption

**Fig. 11:**
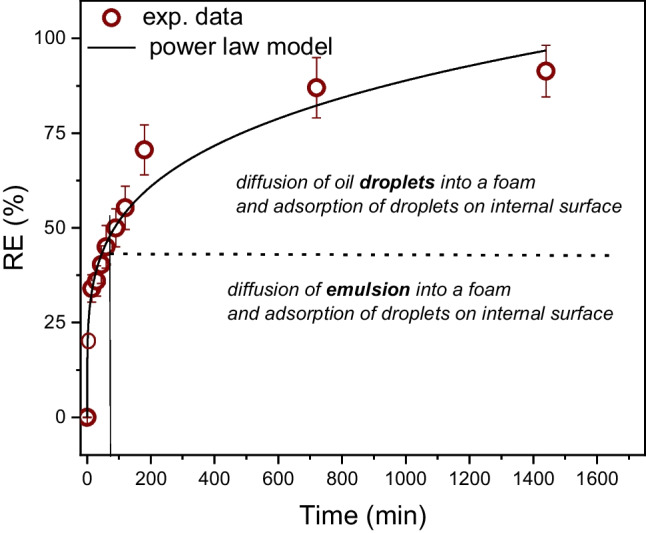
The dependence of removal efficiency (RE) on time

Absorption experiments were performed using seven foams immersed in 50 mL of 100 ppm DO/water emulsion at 22°C, and at various times of sorption in the range from 0 to 24 h. The dependence of the removal efficiency (RE) defined by Eq. (6) on time is shown in Fig. 11.

The whole sorption process can be roughly divided into two stages. In the first stage, the diffusion of the emulsion into foam is a dominant process. It is accomplished by a parallel diffusion of oil droplets within a foam and by the adsorption of oil droplets on the internal surface (fibers) of foam. During this process, a rapid decrease in oil content in the emulsion is observed, resulting in a high rate of removal efficiency. The previous experiment described above indicated that foam is fully saturated after approximately 60 min. In the course of this time, approximately 50% of the initial oil content is removed from the emulsion. However, the removal of oil from the emulsion continues after this time, despite the foam being fully saturated by liquid. A few parallel processes run in this stage. Oily droplets inside a foam continuously diffuse to the fibers and adsorb onto the internal surface of the foam. This leads to a decrease in the local oil concentration within a foam, resulting in the induction of a concentration gradient between the emulsion inside and outside the foam. This effect induces additional transport (diffusion) of oil droplets from the emulsion to the foam. These processes play a significant role for approximately 200 min, and during this period, approximately 73% of oil is removed. Figure 10 shows that after approximately 200 min, the removal of oil from the emulsion starts to significantly decelerate. Over additional time, the adsorption of oil on the MF skeleton decreases, and thus, less oil from the emulsion diffuses into the foam; therefore, the removal efficiency is increasingly decelerated. Finally, when the adsorption of oil droplets on fibers is marginal, the concentrations inside and outside a foam are close to equilibrium, and the process of oil removal from the emulsion stops. In this study, the duration of the whole experiment was arbitrarily chosen as 24 h because the further continuation of the process had a negligible effect on the removal efficiency. The removal efficiency after 24 h was 91.4%.

The most common models describing the adsorption of low molecular weight species from liquids (mostly water) are the pseudo-first-order model (PFOM) (Lagergren 1898), pseudo-second-order model (PSOM) (Ho and McKay 1998), and Weber-Morris intra-particle diffusion model (IPD) (Weber and Morris 1963). In some cases, but not always, these models may also apply to the characterization of absorption processes, as discussed by Khosravi and Azizian (2016). The analysis showed that neither PFOM nor PSOM is suitable for modeling experimental data in this study, first because both these models are derived for monolayer adsorption, and second because the exact amount of adsorbed oil on a skeleton surface of a foam is not known (the part of the oil is incorporated in a residual liquid within a foam after squeezing), and third because the sorption is primarily controlled by diffusion at different time scales. The Weber-Morris intra-particle diffusion model is the most frequently applied model for systems in which adsorption is accomplished by diffusion; however, this model also does not properly fit the presented data due to the non-Fickian character of sorption, as discussed above.

On the other hand, the generalized non-Fickian diffusional model given by Eq. (4) can be adapted for the description of oil sorption within a foam.

The time-dependent sorption capacity (*S*_w_) of a foam can be expressed by Eq. (7):

7 $${S}_{w}\left(t\right)=\frac{{c}_{0}-c(t)}{{m}_{0,f}}V$$

where *c*_0_ [mg/L] is the initial concentration of oil in the emulsion, *c*(*t*) is the concentration of oil in the emulsion at time *t*, *V* [mL] is the volume of emulsion considered for analysis, and *m*_0,f_ [mg] is the initial weight of the neat foam. In equilibrium (in the limit case at *t* → ∞), the concentration of oil in the emulsion is *c*(*t* → ∞), and the limit sorption capacity (*S*_w_^∞^) is given by Eq. (8):

8 $${S}_{w}^{\infty }=\frac{{c}_{0}-c(t\to \infty )}{{m}_{0,f}}V$$

The sorption process can be also characterized by the time-dependent removal efficiency (RE(*t*)) defined by Eq. (9):

9 $$RE\left(t\right) [\%]=\frac{{c}_{0}-c\left(t\right)}{{c}_{0}}\times 100\%; \;{RE}^{\infty }[\%]=\frac{{c}_{0}-c(t\to \infty )}{{c}_{0}}\times 100\%$$

Then, the non-Fickian diffusional model (Eq. (4)) can be rewritten into Eq. (10):

10 $$\frac{RE(t)}{{RE}^{\infty }}=k{t}^{n}$$

Figure 11 shows that this model appropriately fits the experimental data. The fitting function was applied in the general form of RE(*t*) = *At*^*n*^ (*A* = RE^∞^*k*), and the following parameters were found: *A* = 17.0 ± 2.0 [%.min^−*n*^], *n* = 0.24 ± 0.02 [ −], and *R* = 0.99399. Parameter *n* is significantly smaller than 0.5, indicating a strongly non-Fickian character of absorption. The mutual separation of parameters ***k*** and **RE**^**∞**^ from the fitting parameter ***A*** requires some precondition to set one parameter as the constant. For simplicity, RE^∞^ can be roughly estimated from the real experimental value of the oil content in the emulsion at the end of the experiment. In this case, if RE^∞^ is 91.5%, then the rate constant *k* = 0.019 min^−*n*^.

#### The reusability of foams

**Fig. 12:**
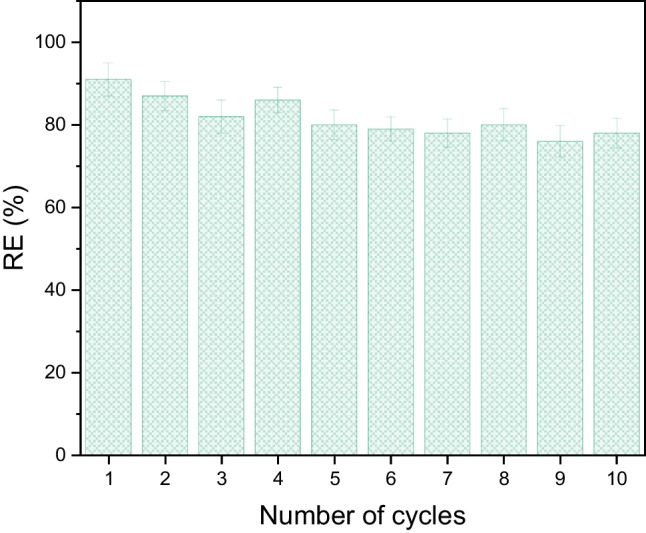
The removal efficiency of MFs for ten constrictive cycles

The regeneration and reusability of sorbents are important points for their practical application in wastewater treatment (Saleem et al. 2021). In the case of foamy sorbents, the easiest method of sorbent recovery is simple squeezing, which is mostly applicable for foam recovery after the sorption of neat oils (Pintor et al. 2016; Hailan et al. 2021). On the other hand, in the case that adsorption sites are saturated and adsorption cannot proceed, the regeneration of sorbents by various thermal, chemical, and microbiological methods must be applied (Salvador et al. 2015a, b). In this study, only the regeneration of MFs through simple squeezing was tested. For this reason, ten constrictive sorption–desorption cycles were performed using an emulsion containing 100 ppm DO (72 ± 4 ppm of the carbon in the emulsion) and seven foams. The duration of each cycle was 4 h. The foams used in the *i*th cycle were the same foams, which were used in the (*i* − 1)^th^ cycle; the only liquid was squeezed out, and no additional drying or cleaning treatment was performed. The removal efficiency of MFs for ten cycles is shown in Fig. 12.

The experiments found that the efficiency of the foams slightly decreases with an increased number of cycles due to oil sorption on the foam skeleton that cannot be removed by simple squeezing of the sponge. Additionally, a simple squeezing as performed in this study cannot remove the whole liquid from the foam. Quantitatively, the mass of the foams is 0.191 ± 0.004 g, the mass of the absorbed liquid is 17.4 ± 0.6 g, and the mass of the permanently trapped liquid is 1.37 ± 0.08 g; thus, the permanently trapped liquid after squeezing is approximately 8% of the absorbed liquid. On the other hand, the trapped liquid content does not increase with an increase in the number of cycles and remains more or less constant.

### Separation of highly concentrated oil in water mixtures

**Fig. 13:**
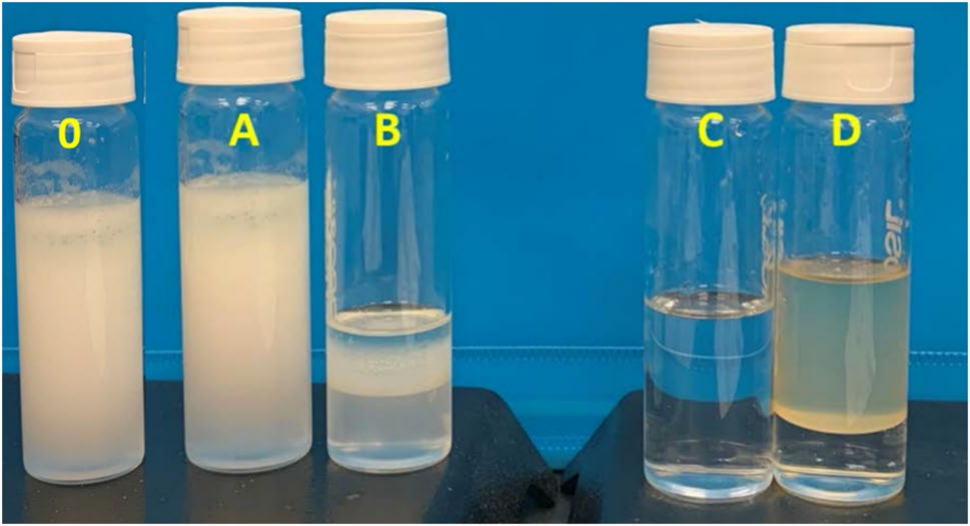
Photographs of the initial o/w mixture (40/60 w/w). O, initial mixture; A, the emulsion was purified by neat, untreated MFs—a filtrate; B, the emulsion was purified by neat, untreated MFs—a liquid that was squeezed out of the foams; C, the emulsion was purified by treated MFs—a filtrate; D, the emulsion was purified by treated MFs—a liquid that was squeezed out of the foams

This paragraph is focused on the separation of highly concentrated oil/water mixtures composed of large portions of both components (o/w = 20/80, 40/80 w/w). The concept is based on different rates of diffusion of oil and water into the treated foams due to the changed MF wettability, as discussed above. The mixtures of DO/distilled water (20/80, 40/60 w/w) were prepared by vigorous mechanical stirring, and the sorption experiment was performed immediately after mixing using seven treated MFs immersed into 50 mL of the mixture in a Falcon tube (shaken) in room temperature (22 °C) for 4 h. During the process, DO penetrates the foams, whereas water rests in the tube. At the end of the experiment, the residual liquid (filtrate) was taken from the tube, sonicated to ensure homogeneity of the mixture, and TOC was determined. The experiment was repeated four times. The carbon content in the filtrate was found to be 274 ± 42 ppm for the 20/80 mixture and 481 ± 76 ppm for the 40/60 w/w mixture, indicating exceptionally high removal efficiencies of 99.86% and 99.88%, respectively. The separation efficiency of the treated MFs is clearly shown in Fig. 13. For comparison, untreated MFs were tested under the same conditions. In this case, the filtrate contained a high amount of oil, and it tended to phase separately after shaking was stopped. The mixture was kept at rest for 1 h, the top oil layer was removed, and the residual liquid was sonicated and tested for carbon content. The carbon content in the filtrate was over 9000 ppm for the 20/80 w/w mixture. The microstructure after the separation was studied by profilometry and showed negligible changes in roughness with a Sa value of 147.4 µm ± 12.4 µm (Fig. S1 ESI).

**Fig. 14:**
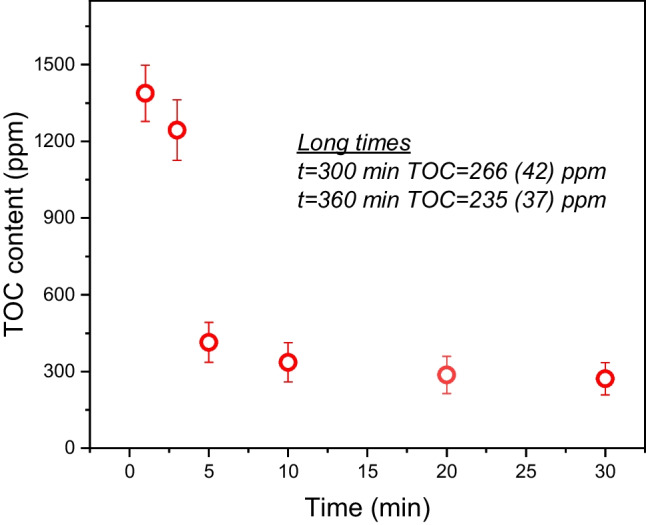
TOC content in the filtrate as a function of treatment time

In the second step, the kinetics of sorption were tested for the 20/80 w/w mixture only under the same conditions. The sorption experiment was individually performed for each selected time of immersion (1, 3, 5, 10, 20, 30, 300, and 360 min). The dependence of the carbon content in filtrates versus time of immersion is shown in Fig. 14. The longest times are not included in the graph for practical reasons to keep the scale to a reasonable extent. The results indicate that the oil sorption into the foams is very fast and that the majority of oil penetrates the foam within minutes, and after 5 min, the changes in oil concentration in the filtrate with additional time are minimal.

**Fig. 15:**
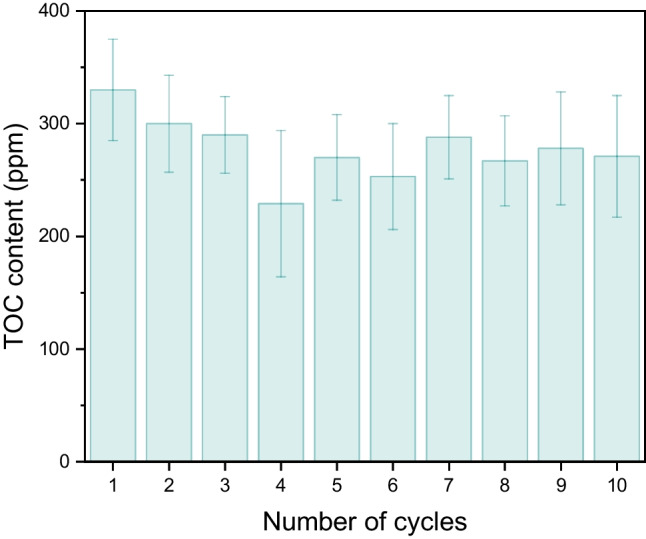
TOC content in the filtrate as a function of the number of consecutive constrictive cycles

Finally, the reusability of MFs was tested by performing ten constrictive separation cycles. The duration of each cycle was 4 h. The foams used in the *i*th cycle were the same foams used in the (*i* − 1)^th^ cycle immediately after squeezing, and no additional treatment, such as drying or cleaning, was performed. The experiment found that the removal efficiency of the foams did not change significantly over cycling, and the TOC values were less constant within a range given by measurement standard deviations (Fig. 15).

### Removal of free oil from the water surface

The removal of free oil from the water surface is the most commonly investigated application of porous, hydrophobic, and oleophilic materials in water treatment. Such materials absorb oil to a high extent because of their high porosity (nonswelling thermosetting polymers) or due to a combination of porosity and swelling ability (rubber-like materials). Melamine and polyurethane foams are among the most investigated materials for this purpose (Hailan et al. 2021). This is also the simplest applicability of modified MFs in this study. Here two different situations may happen. Firstly, foams are in contact with oil only, without any touch with water. In this case, both treated and untreated foams rapidly absorb oil, proportionally to their porosity, and the treatment (an enhanced hydrophobicity) does not bring any additional benefit because of the oleophilicity of both untreated and treated foams. On the other hand, a situation is quite different if foams can get in touch with water, e.g., if free oil floats on the water’s surface and foams are used for its removal. In this case, hydrophobic treatment of foam is needed, because sorption of both oil and water are competitive processes running in a parallel way. It was shown that hydrophobic treatment of MFs almost completely suppresses the absorption of water in case foams are floating on the water’s surface.

**Fig. 16:**
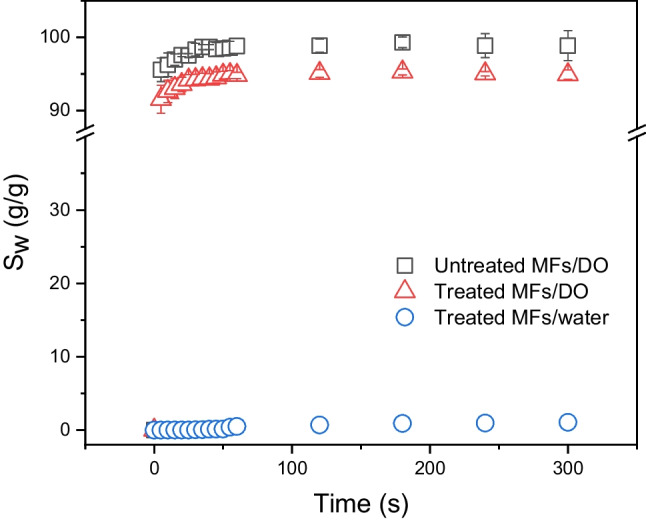
The dependence of uptake capacity (*S*_w_) on time

The dependences of the uptake capacity (*S*_w_ = mass of absorbed DO/mass of pristine foam) of foams on time are shown in Fig. 16. For comparison, both untreated and treated MFs were tested. Both foams absorb a large amount of DO in a very short time, on the order of seconds. The uptake capacity of treated MFs determined after 5 min of DO exposure is 95 g/g, and the uptake capacity of untreated foam is slightly higher, 99 g/g, which can be the result of slightly higher porosity. The core difference between untreated and treated MFs lies in their completely different sorption of water. Whereas untreated MF immediately absorbs water to a large extent, treated MF absorbs water only very slightly. In this experiment, the foam floating on the water surface for 300 s showed a water uptake of approximately 2.1 g/g. This observation does not contradict the results reported above for water sorption into immersed foams, as these two cases are different. In the case of foam floating on the water surface, there are no sufficiently high forces that can push water into pores or voids on the external surface of foam, and these pores, initially filled by air, can be only slightly refilled by water, unlike fully immersed foams.

**Fig. 17:**
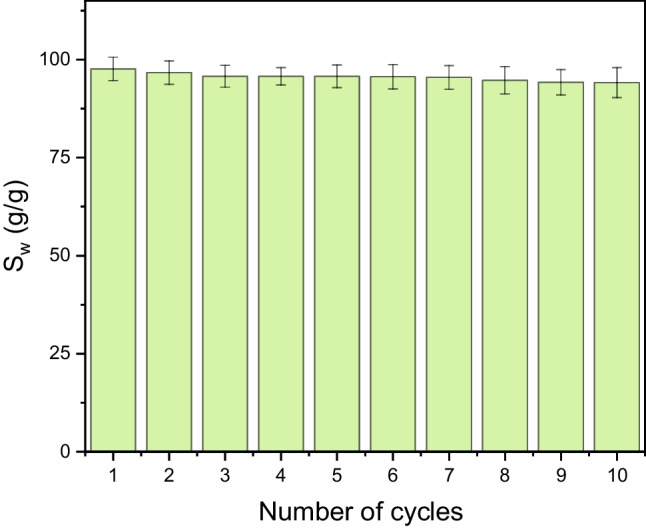
The uptake capacity (*S*_w_) of treated MFs as a function of the number of constrictive cycles

Finally, multiple uses of treated MFs were demonstrated through repeating sorption–desorption cycles (Fig. 17). After each cycle, oil was squeezed out of the foam in a syringe, and the foam was used in the next cycle without any additional treatment. For illustration, only ten cycles were performed in this study; however, it has been reported in the literature that melamine foam can be used for multiple oil sorption more than thousands of times (Zhou et al. 2016; Ruan et al. 2014).

## Conclusion

This work demonstrates the efficient, low-cost, and scalable treatment of oily polluted waters including colloidal emulsions, oil-in-water mixtures, and free oil removal using melamine foams (MFs) modified by ferric chloride.

To our best knowledge, this is the first study reporting such broadly applicable single material for a complex purification of oil-in-water systems including low-concentrated colloidal waters, unlike a majority of studies focused on the hydrophobization of foams targeted mostly for free oil removal or highly concentrated mixtures, as summarized in Table S1 in ESI1. Diesel oil was used as a reasonable, easily available model of the hydrocarbon fractions occurring in refinery wastewaters or industrially produced waters instead of various grades of crude oil.

The emulsions containing 100 ppm DO content were separated with 91.4% efficiency, and the mixtures containing 20 and 40 vol. % DO were separated with 99.9% efficiency. Modified foams also rapidly remove free DO from the water surface, absorbing 95 g/g DO, whereas water sorption is negligible. Multiple usages of these foams for all these niche operations were also proven. The most important output of this study is a demonstration of an efficient demulsification of low-concentrated colloidal emulsions by modified MF foam. The mechanism is based on multiple diffusion processes running at different time scales, including diffusion of the emulsion into the foam, diffusion of oil droplets within the foam, and diffusion of individual oil droplets into the foam combined with parallel adsorption of oil droplets on the solid skeleton of the foam. Ferric chloride, which was used to change the wettability of MFs from hydrophilic to hydrophobic, also contributes to the adsorption of negatively charged species.

This work indicates that melamine foams treated by FeCl_3_ represent a simple and cheap approach to the treatment of emulsified water containing oily impurities in a colloidal state, as well as for a separation of o/w mixtures and free oil spill removal.

## Supplementary Information

Below is the link to the electronic supplementary material.

Supplementary file1 (M4V 311692 KB)

Supplementary file2 (DOCX 107 KB)

## Data Availability

The data that support the findings of this study are openly available on request.

## References

[CR1] Abu-Thabit NY, Uwaezuoke OJ, Abu Elella MH (2022). Superhydrophobic nanohybrid sponges for separation of oil/ water mixtures. Chemosphere.

[CR2] Adham S, Hussain A, Minier-Matar J (2018). Membrane applications and opportunities for water management in the oil & gas industry. Desalination.

[CR3] Aguilera F, Méndez J, Pásaroa E, Laffona B (2010). Review on the effects of exposure to spilled oils on human health. J Appl Toxicol.

[CR4] Al-Ghouti MA, Al-Kaabi MA, Ashfaq MY, Da’na DA (2019). Produced water characteristics, treatment and reuse: a review. J. Water Process Eng..

[CR5] Albatrni H, Qiblawey H, Almomani F (2019). Polymeric adsorbents for oil removal from water. Chemosphere.

[CR6] Cassie ABD, Baxter S (1944). Wettability of porous surfaces. Trans Faraday Soc.

[CR7] Chen S, He G, Hu H (2013). Elastic carbon foam via direct carbonization of polymer foam for flexible electrodes and organic chemical absorption. Energy Environ Sci.

[CR8] Dąbrowski A (2001). Adsorption — from theory to practice. Adv Colloid Interface Sci.

[CR9] Dardor D, Al-Maas M, Minier-Matar J (2021). Protocol for preparing synthetic solutions mimicking produced water from oil and gas operations. ACS Omega.

[CR10] Dashairya L, Gopinath M, Saha P (2020) Synergistic effect of Zr/Cl dual-ions mediated pyrrole polymerization and development of superhydrophobic melamine sponges for oil/water separation. Colloids Surf A Physicochem Eng Asp 599. 10.1016/J.COLSURFA.2020.124877

[CR11] Dickhout JM, Moreno J, Biesheuvel PM (2017). Produced water treatment by membranes: a review from a colloidal perspective. J Colloid Interface Sci.

[CR12] Ding Y, Xu W, Yu Y (2018). One-step preparation of highly hydrophobic and oleophilic melamine sponges via metal-ion-induced wettability transition. ACS Appl Mater Interfaces.

[CR13] Ding Y, Xu W, Yu Y et al (2018b) One-step preparation of highly hydrophobic and oleophilic melamine sponges via metal-ion-induced wettability transition. 10.1021/acsami.7b13626 10.1021/acsami.7b13626 29376631

[CR14] Diraki A, Mackey HR, McKay G, Abdala A (2019). Removal of emulsified and dissolved diesel oil from high salinity wastewater by adsorption onto graphene oxide. J Environ Chem Eng.

[CR15] Fredrick E, Walstra P, Dewettinck K (2010). Factors governing partial coalescence in oil-in-water emulsions. Adv Colloid Interface Sci.

[CR16] Gulyas H, Reich M (1995). Organic compounds at different stages of a refinery wastewater treatment plant. Water Sci Technol.

[CR17] Hailan SM, Ponnamma D, Krupa I (2021). The separation of oil/water mixtures by modified melamine and polyurethane foams: a review. Polymers (basel).

[CR18] Han L, Khalil AME, Wang J (2021). Graphene-boron nitride composite aerogel: a high efficiency adsorbent for ciprofloxacin removal from water. Sep Purif Technol.

[CR19] Han L, Li X, Li F (2022). Superhydrophilic/air-superoleophobic diatomite porous ceramics for highly-efficient separation of oil-in-water emulsion. J Environ Chem Eng.

[CR20] Han L, Wu W, Huang Z (2021). Preparation and characterization of a novel fluorine-free and pH-sensitive hydrophobic porous diatomite ceramic as highly efficient sorbent for oil–water separation. Sep Purif Technol.

[CR21] Ho YS, McKay G (1998). The kinetics of sorption of basic dyes from aqueous solution by sphagnum moss peat. Can J Chem Eng.

[CR22] Khosravi M, Azizian S (2016). A new kinetic model for absorption of oil spill by porous materials. Microporous Mesoporous Mater.

[CR23] Lagergren S (1898). Zur theorie der sogenannten adsorption geloster stoffe. K Sven Vetenskapsakademiens Handl.

[CR24] Li J, Li D, Hu W (2015). Stable superhydrophobic and superoleophilic silica coated polyurethane sponges for the continuous capture and removal of oils from the water surface. New J Chem.

[CR25] Li X, Han L, Huang Z (2022). A robust air superhydrophilic/superoleophobic diatomite porous ceramic for high-performance continuous separation of oil-in-water emulsion. Chemosphere.

[CR26] Neff J, Lee K, DeBlois EM (2011). Produced water: overview of composition, fates, and effects. Produced water.

[CR27] Oliveira LMTM, Saleem J, Bazargan A (2021). Sorption as a rapidly response for oil spill accidents: a material and mechanistic approach. J Hazard Mater.

[CR28] Ong C, Shi Y, Chang J (2019). Polydopamine as a versatile adhesive layer for robust fabrication of smart surface with switchable wettability for effective oil/water separation. Ind Eng Chem Res.

[CR29] Patterson JW, Patterson JW (1985) Industrial wastewater treatment technology. 2nd Edition, Butterworth Publishers, Stoneham

[CR30] Pinto J, Athanassiou A, Fragouli D (2018). Surface modification of polymeric foams for oil spills remediation. J Environ Manage.

[CR31] Pintor AMA, Vilar VJP, Botelho CMS, Boaventura RAR (2016). Oil and grease removal from wastewaters: sorption treatment as an alternative to state-of-the-art technologies. A critical review. Chem Eng J.

[CR32] Qin P, Chen D, Li M et al (2022) Melamine/MIL-101(Fe)-derived magnetic carbon nanotube-decorated nitrogen-doped carbon materials as sorbent for rapid removal of organic dyes from environmental water sample. J Mol Liq 359. 10.1016/J.MOLLIQ.2022.119231

[CR33] Rahman SS (1992). Evaluation of filtering efficiency of walnut granules as deep-bed filter media. J Pet Sci Eng.

[CR34] Ritger PL, Peppas NA (1987). A simple equation for description of solute release I. Fickian and non-fickian release from non-swellable devices in the form of slabs, spheres, cylinders or discs. J Control Release.

[CR35] Ritger PL, Peppas NA (1987). A simple equation for description of solute release II. Fickian and anomalous release from swellable devices. J Control Release.

[CR36] Ruan C, Ai K, Li X, Lu L (2014). A superhydrophobic sponge with excellent absorbency and flame retardancy. Angew Chemie Int Ed.

[CR37] Saleem J, Dotto GL, McKay G (2021). Current scenario and challenges in using plastic wastes as oil absorbents. J Environ Chem Eng.

[CR38] Salvador F, Martin-Sanchez N, Sanchez-Hernandez R (2015). Regeneration of carbonaceous adsorbents. Part I: thermal regeneration. Microporous Mesoporous Mater.

[CR39] Salvador F, Martin-Sanchez N, Sanchez-Hernandez R (2015). Regeneration of carbonaceous adsorbents. Part II: chemical, microbiological and vacuum regeneration. Microporous Mesoporous Mater.

[CR40] Stuart BH (2004). Infrared spectroscopy: fundamentals and applications.

[CR41] Takahashi M, Sakamoto K (2017) Regulations on cosmetics. In: Cosmetic science and technology. Elsevier, pp 137–146. 10.1016/b978-0-12-802005-0.00009-4

[CR42] Tanudjaja HJ, Hejase CA, Tarabara VV (2019). Membrane-based separation for oily wastewater: a practical perspective. Water Res.

[CR43] Vásquez L, Campagnolo L, Athanassiou A, Fragouli D (2019). Expanded graphite-polyurethane foams for water-oil filtration. ACS Appl Mater Interfaces.

[CR44] Wang N, Wang Y, Shang B (2018). Bioinspired one-step construction of hierarchical superhydrophobic surfaces for oil/water separation. J Colloid Interface Sci.

[CR45] Weber WJ, Morris JC (1963). Kinetics of adsorption on carbon from solution. J Sanit Eng Div.

[CR46] Wenzel RN (1936). Resistance of solid surfaces to wetting by water. Ind Eng Chem.

[CR47] Yin X, Zhang J, Wang X, Zhu M (2020) Modified walnut shell filter material for the enhanced removal of oil from oilfield wastewater. Environ Eng Res 26. 10.4491/eer.2019.369

[CR48] Zhang W, Wang J, Han X (2021). Carbon nanotubes and polydopamine modified poly(dimethylsiloxane) sponges for efficient oil–water separation. Materials (basel).

[CR49] Zhang XF, Song L, Chen X (2020). Zirconium ion modified melamine sponge for oil and organic solvent cleanup. J Colloid Interface Sci.

[CR50] Zhou L, Wang X, Yuan K (2016). Mussel-inspired fabrication of novel superhydrophobic and superoleophilic sponge modified using a high density of nanoaggregates at low concentration of dopamine. RSC Adv.

