# Supplementary material for: Complex treatment of oily polluted waters by modified melamine foams: from colloidal emulsions to a free oil removal
Source: Environ Sci Pollut Res Int. 2023 Aug 21;30(43):97872–87. doi: 10.1007/s11356-023-29055-x (PMC10495526; doi:10.1007/s11356-023-29055-x)
Supplement: Supplementary file 2 — Supplementary file2 (DOCX 107 KB) [file 11356_2023_29055_MOESM2_ESM.docx]

**Supporting Information**

**Complex treatment of oily polluted waters by modified melamine foams: From colloidal emulsions to a free oil removal**

Sarah Hailan^a^, Patrik Sobolciak^a^, Anton Popelka^a^, Peter Kasak^a^, Samer Adham^b^, and Igor Krupa^a*^

^a^ Center for Advanced Materials, Qatar University, P. O. Box 2713, Doha, Qatar

^b^ ConocoPhillips Global Water Sustainability Center, Qatar Science, and Technology Park, P. O. Box 24750, Doha, Qatar

(^*^corresponding author, igor.krupa@qu.edu.qa)

**Figure S1:** Representative profilometer micrograph of modified MF after oil sorption experiments.


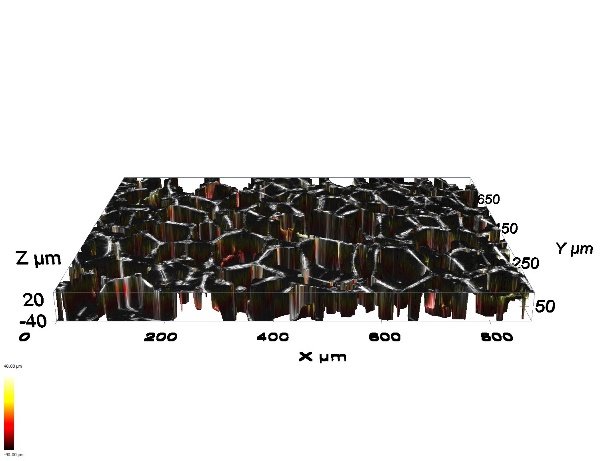

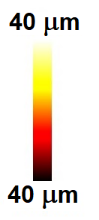


**Table S1**. Modified melamine foams (MF) and polyurethane foams (PU) recently reported in the literature with their corresponding maximum water contact angle (WCA°), gravimetric absorption capacity range (AC) in g/g, oil/ water (o/w) separation ability, and demulsification process including surfactant-stabilized oil in water emulsion (SOWE), surfactant-stabilized water in oil emulsion (SWOE), surfactant-free oil in water emulsion (OWE) and surfactant-free water in oil emulsion (WOE).

| **F** | **Treatment** | **WCA** **(°)** | **AC (g/g)** | **o/w mixture separation** | **Demulsification process** | **Ref** |
| --- | --- | --- | --- | --- | --- | --- |
| **MF** | FeCl_3_ | Max. 153° | 95 g/g for diesel | Oil type: diesel oil  o/w mixture initial ratio:  (20/80 and 40/60 w/w)  Water purity after oil removal:  The total organic carbon analyzer (TOC) indicated an oil concentration of 274±42  ppm for the 20/80 mixture and 481±76 ppm for the 40/60 w/w mixture which reflect removal efficiencies of 99.86% and 99.88%, respectively.  Additional information**:** o/w mixtures separation process was done by mechanical steering for 10 minutes. | Emulsion type: OWE  Diesel oil + water  Emulsion concentration: 100 ppm  Characterization method: TOC was used to determine the emulsion and the filtrate concentration. The resultant efficiency is 91.4%  Additional information: Modified foams were added to the emulsion and mechanically stirred for 24 h | This work |
| MF | FeCl_3_ (0.0005-0.1M), Fe(NO_3_)_3_, Zn(NO_3_)_2_, Ni(NO_3_)_2_  and Co(NO_3_)_2_) | 130**°** for 0.01M-FeCl_3_/MF sample | 71 g/g for n-hexane -157 g/g for chloroform ( 0.1M- FeCl_3_/MF sample) | NA | No demulsification experiment was performed | 1 |
| MF | Polybenzoxazine | 162**°** | 62 g/g for n-hexane -170 g/g for chloroform | Oil type: dichloromethane  o/w mixture initial ratio: not reported  Water purity: not reported.  Additional information: | Emulsion type: SOWE  Toluene (2 ml) + water (200 ml) + Span 80 surfactant (0.3 g)  Emulsion concentration: not reported  Characterization method: dynamic light Scattering (DLS) device showed a droplet size change from 2480 ± 410 nm to 58.8 ± 4.9 nm for the gravity-driven separation process and to 99.7 ± 9.4 nm for the pressure-driven separation process  Additional information: gravity-driven and pressure-driven (0.025 bar) separation processes were applied | 2 |
| MF | C_9_H_8_O_4_-PEI | 107**°** | NA | NA | Emulsion type: SOWE  Different oils including, gasoline, n-hexane, toluene, n-decane, motor, octadecane, and petroleum ether oil (2 ml) + water (200 ml) + Tween 80 surfactant (0.02 g)  Emulsion concentration: not reported  Characterization method: DLS showed that oil droplet size changed from 1000–2000 nm down to 100 nm  Additional information: homemade dead-end filtration setup was applied | 3 |
| MF | DA/ PEI | 140**°** | 67 g/g for n-hexane -179 g/g for trichloromethane | Oil type: paraffin oil  o/w mixture initial ratio: not reported  Water purity: not reported  Additional information: | Emulsion type: SOWE  Paraffin oil (2 g) + water (198 ml) + different surfactants including, SDBS, Brij-35 , Span-80, and CTAB (0.2 g)  Emulsion concentration: not reported  Characterization method: DLS and infrared oil content analyzer were used to determine the foam efficiency by oil content in emulsion and filtrate and the determined efficiencies were 59% for Span-80, 27% for Brij-35, and 15 % for CTAB OWE emulsion  Additional information: separation process was comprised of DA/PEI foam, a needleless syringe, and a vacuum system. | 4 |
| MF | rGO | 164**°** | 59 g/g for crude oil – 150 g/g for chloroform | Oil type: shell oil, pump oil, and crude oil  o/w mixture initial ratio: 2:4 v/v  Water purity: not reported  Additional information: | Emulsion type: OWE  Pump oil + water ( 30 g/l)  Emulsion concentration: not reported  Characterization method: Chemical oxygen demand (COD) was used to determine the emulsion and filtrate concentration and accordingly the foam performance was 92% at room temperature  Additional information: demulsification was done by stirring emulsion containing foams | 5 |
| MF | β-FeOOH nanoparticles | 153**°** | 65 g/g for kerosene - 136 g/g for chloroform | Oil type: dodecane  o/w mixture initial ratio: 1:3 v/v  additional information:  -vacuum-assisted o/w separation process was applied  -efficiency by volume was determined to be 98% | Emulsion type: OWE  Dodecane + water (1: 50 v/v)  Emulsion concentration: 2949.59 ppm  Characterization method: TOC showed a filtrate concentration of 20.78 ppm  Additional information: emulsion containing foams undergo agitation for 30 min | 6 |
| MF | Mg(OH)_2_ | 160**°** | 70 g/g for kerosene oil – 190 g/g for carbon tetrachloride | NA | Emulsion type: SOWE  Toluene + water (1:2) + CTAB and SDS surfactants  Emulsion concentration: not reported  Characterization method: DLS showed oil particle size decrease from 5 μm to around 2.4 μm for nearly all emulsions  Additional information: homemade separation setup using a prelatic pump was used | 7 |
| MF | Co-ZIF-L | NA | NA | Oil type: hexane  o/w mixture initial ratio: 1:1 v/v  Water purity: not reported  Additional information: gravity-driven o/w separation process was applied | Emulsion type: SOWE  n-dodecane + water (1:100 w/w) + SDS surfactant (200 mg)  Emulsion concentration: not reported  Characterization method: UV-Vis Spectroscopy (the initial concentration of emulsion and filtrate concentration were not reported)  Additional information: gravity-based separation process was applied | 8 |
| MF | PDA/DDT | NA | 70 g/g for methanol - 99 g/g for chloroform | Oil type: toluene  o/w mixture initial ratio: not reported  Water purity: not reported  Additional information:vacuum-assisted o/w separation process was applied | Emulsion type: OWE  Chloroform + water (1:10 v/v)  Emulsion concentration: not reported  Characterization method: UV-Vis Spectroscopy showed a transparency of 91%% for the filtrate  Additional information: demulsification was done by stirring emulsion containing foams | 9 |
| MF | PVDF-HFP+Fe_3_O_4_ NPs | ~130**°** | 29 g/g for petroleum ether - 44 g/g for toluene | NA | Emulsion preparation: OWE  Toluene + water (1:10 v/v)  Emulsion concentration: not reported  Characterization method: physical observation of a transparent filtrate  Additional information: demulsification was done by stirring emulsion containing foams | 10 |
| MF | 1H, 1H, 2H, 2H-PFDT | 154**°** | 17 g/g for petroleum ether - 34 g/g for tetrachloromethane | Oil type: tetrachloromethane and n-hexane  o/w mixture initial ratio: 1:1 v/v  Water purity: not reported  Additional information: | Emulsion type: SOWE & OWE  Tetrachloromethane oil + water (1:30 v/v) + SDS surfactant  Emulsion concentration: not reported  Characterization method: optical light microscopy was used to observe the filtrate and less amount of oil was observed compared to the initial emulsion  Additional information: demulsification was done by stirring emulsion containing foams | 11 |
| PU | Stearic acid | 151**°** | 17 g/g to 42 g/g for tetrahydrofuran | Oil type: gasoline and diesel oil  o/w mixture initial ratio: not reported  Water purity: not reported  Additional information: vacuum-assisted o/w separation process was applied | Emulsion type: OWE  Toluene oil + water (1:10 v/v)  Emulsion concentration: not reported  Characterization method: UV-Vis Spectroscopy showed a transparency of 80%% for the filtrate  Additional information: demulsification was done by stirring emulsion containing foams | 12 |
| PU | 3,5-bis(trifluoromethyl) benzene diazonium tosylate  [ADT-(CF_3_)_2_] | 168**°** | 40 g/g for petroleum ether - 75 g/g for chloroform | Oil type: chloroform (20ml)  o/w initial ratio: not reported  Water purity: not reported  Additional information: | Emulsion type: WOE & SWOE  pump and rapeseed oil + water ( 1:99) + Pluronic F-127 surfactant  Emulsion concentration: not reported  Characterization method: oil purity by an extractive-gravimetric method using n-hexane as a solvent was determined to be 99%.  Additional information:: demulsification was done by stirring emulsion containing foams | 13 |
| PU | 1,3-oxazolidine/ 1,4-dioxane, stearoyl chloride, and NaHCO_3_ | 152**°** | 17 g/g for petroleum ether and n-hexane - and 40 g/g for chloroform | Oil type: crude oil  o/w mixture initial ratio: not reported  Water purity: not reported  Additional information: | Emulsion type: OWE  Crude oil + water (1:9 v/v)  Emulsion concentration: not reported  Characterization method physical observation of a transparent filtrate  Additional information: demulsification was done by stirring emulsion containing foams | 14 |
| PU | carbon cloth (CC)/ZnO/SA | 160**°** | 40g/g for engine oil-70 g/g for kerosene oil | Oil type: kerosene  o/w mixture initial ratio: not reported  Water purity: not reported  Additional information: | Emulsion preparation: OWE  Toluene oil + water (ratio not reported)  Emulsion concentration: not reported  Characterization method: optical light microscopy was used to observe the filtrate and less amount of oil was observed compared to the initial emulsion  Additional information: emulsion containing foams undergo agitation for a certain time | 15 |
| PU | octadecyltrichlorosilane (OTS) | 156**°** | 22 g/g for n-hexane – 25 g/g for bean oil | Oil type: bean oil  o/w mixture initial ratio: not reported  Water purity: not reported  Additional information: | Emulsion type: SOWE  Toluene + water emulsion (ratio not reported)  Emulsion concentration: not reported  Characterization method: DLS of filtrate oil droplets was 710 nm, however, the DLS of the initial emulsion was not reported  Additional information: demulsification was done by stirring emulsion containing foams | 16 |
| PU | PDA/Fe_3_O_4_/Ag | 156**°** | 23g/g for petroleum ether –52 g/g for chloroform. | NA | Emulsion type: SOWEs  Toluene + water emulsion (1:19 v/v) + Pluronic F-127 surfactant (0.1 mg/l)  Emulsion concentration: not reported  Characterization method: DLS showed droplet decrease from 1805 nm to 18006 nm before separation down to 475 nm to 1075 nm after separation  Additional information: demulsification was done by stirring emulsion containing foams | 17 |
| PU | Graphene | 152**°** | NA | Oil type: engine, crude, and hexane  o/w mixture initial ratio: not reported  water purity: not reported  additional information: oil collecting device composed of a modified sponge, a glass bottle under pressure (30kPa), and a collecting vessel was used | Emulsion type: SOWE  Different oils including, hexane, hexadecane, and soybean oil + water (ratio not reported) + Tween 80 surfactant  Emulsion concentration: not reported  Characterization method: Infrared oil-content analyzer revealed filtrate concentrations of 19.92, 13.78, and 17.98 ppm for hexane, hexadecane, and soybean oil/ water emulsions  Additional information: demulsification was done by stirring emulsion containing foams | 18 |
| PU | MCFO/rGO nanocomposite | 165**°** | 40 g/g for cooked oil–131 g/g for chloroform | Oil type: dichloromethane (20ml)  o/w mixture initial ratio: not reported  water purity: not reported  additional information: funnel supported o/w separation process was used | Emulsion type: OWE & SOWE  Toluene oil and water emulsion (1:9 v/v) and Pluronic F-127 surfactant (0.1 mg/l)  Emulsion concentration: not reported  Characterization method: UV-Vis Spectroscopy showed a reduction in the toluene content from 90% to 0.1% in the OWE as well as from 10% to 0.01% for the SOWE  Additional information: demulsification was done by stirring emulsion containing foams | 19 |
| PU | DA+ fly  ash, and dodecanethiol | 161**°** | 34 g/g for kerosene–47 g/g for chloroform | NA | Emulsion type: OWE  Different oils including, n-hexane, toluene, chloroform, kerosene, gasoline, and diesel + water (1:10)  Emulsion concentration: not reported  Characterization method: reported efficiency of 93% as analyzed by infrared spectrometer oil content analyzer, however, emulsion and filtrate concentration were not reported  Additional information: demulsification was done by stirring emulsion containing foams | 20 |
| PU | HDPE/ Fe_3_O_4_ | 155 | 40-75 | Oil type: toluene, hexane, chloroform and carbon tetrachloride  o/w mixture initial ratio: not reported  Water purity: not reported  Additional information: funnel supported o/w separation process was applied | Emulsion type: OWE & SOWE  Toluene + water (1:9 v/v) + Pluronic F-127 surfactant (0.1 mg/l)  Emulsion concentration: not reported  Characterization method: UV-Vis Spectroscopy showed a reduction of toluene content from 10% to 0.18%  Additional information: demulsification was done by stirring emulsion containing foams | 21 |

***NA indicates that this section was not included in the manuscript**

**Movie S1:**

**References**

1. Ding, Y.; Xu, W.; Yu, Y.; Hou, H.; Zhu, Z. One-Step Preparation of Highly Hydrophobic and Oleophilic Melamine Sponges via Metal-Ion-Induced Wettability Transition. *ACS Appl. Mater. Interfaces* **2018**, *10*, 6652–6660, doi:10.1021/acsami.7b13626.
2. Ejeta, D.D.; Wang, C.F.; Lin, C.H.; Kuo, S.W.; Chen, J.K.; Tsai, H.C.; Hung, W.S.; Hu, C.C.; Lai, J.Y. Preparation of a main-chain-type polybenzoxazine-modified melamine sponge via non-solvent-induced phase inversion for oil absorption and very-high-flux separation of water-in-oil emulsions. *Sep. Purif. Technol.* **2021**, *263*, 118387, doi:10.1016/j.seppur.2021.118387.
3. Krishnamoorthi, R.; Anbazhagan, R.; Tsai, H.C.; Wang, C.F.; Lai, J.Y. Preparation of caffeic acid-polyethyleneimine modified sponge for emulsion separation and dye adsorption. *J. Taiwan Inst. Chem. Eng.* **2021**, *118*, 325–333, doi:10.1016/j.jtice.2020.12.029.
4. Liu, W.; Huang, X.; Peng, K.; Xiong, Y.; Zhang, J.; Lu, L.; Liu, J.; Li, S. PDA-PEI copolymerized highly hydrophobic sponge for oil-in-water emulsion separation via oil adsorption and water filtration. *Surf. Coatings Technol.* **2021**, *406*, 126743, doi:10.1016/j.surfcoat.2020.126743.
5. Ahmed, R.M.G.; Anis, B.; Khalil, A.S.G. Facile surface treatment and decoration of graphene-based 3D polymeric sponges for high performance separation of heavy oil-in-water emulsions. *J. Environ. Chem. Eng.* **2021**, *9*, 105087, doi:10.1016/j.jece.2021.105087.
6. Chen, G.; Cao, Y.; Ke, L.; Ye, X.; Huang, X.; Shi, B. Plant polyphenols as multifunctional platforms to fabricate three-dimensional superhydrophobic foams for oil/water and emulsion separation. *Ind. Eng. Chem. Res.* **2018**, *57*, 16442–16450, doi:10.1021/acs.iecr.8b03953.
7. Liu, S.; Zhang, Q.; Fan, L.; Wang, R.; Yang, M.; Zhou, Y. 3D Superhydrophobic Sponge Coated with Magnesium Hydroxide for Effective Oil/Water Mixture and Emulsion Separation. *Ind. Eng. Chem. Res.* **2020**, *59*, 11713–11722, doi:10.1021/acs.iecr.0c01276.
8. Cao, M.; Feng, Y.; Chen, Q.; Zhang, P.; Guo, S.; Yao, J. Flexible Co-ZIF-L@melamine sponge with underwater superoleophobicity for water/oil separation. *Mater. Chem. Phys.* **2020**, *241*, 122385, doi:10.1016/j.matchemphys.2019.122385.
9. Wang, J.; Wang, H.; Geng, G. Highly efficient oil-in-water emulsion and oil layer/water mixture separation based on durably superhydrophobic sponge prepared via a facile route. *Mar. Pollut. Bull.* **2018**, *127*, 108–116, doi:10.1016/J.MARPOLBUL.2017.11.060.
10. Li, J.; Tenjimbayashi, M.; Zacharia, N.S.; Shiratori, S. One-Step Dipping Fabrication of Fe3O4/PVDF-HFP Composite 3D Porous Sponge for Magnetically Controllable Oil-Water Separation. *ACS Sustain. Chem. Eng.* **2018**, *6*, 10706–10713, doi:10.1021/acssuschemeng.8b02035.
11. Wang, N.; Deng, Z. Synthesis of magnetic, durable, and superhydrophobic carbon sponges for oil/water separation. *Mater. Res. Bull.* **2019**, *115*, 19–26, doi:10.1016/J.MATERRESBULL.2019.03.007.
12. Wang, J.; Zheng, Y. Oil/water mixtures and emulsions separation of stearic acid-functionalized sponge fabricated via a facile one-step coating method. *Sep. Purif. Technol.* **2017**, *181*, 183–191, doi:10.1016/j.seppur.2017.03.024.
13. Guselnikova, O.; Barras, A.; Addad, A.; Sviridova, E.; Szunerits, S.; Postnikov, P.; Boukherroub, R. Magnetic polyurethane sponge for efficient oil adsorption and separation of oil from oil-in-water emulsions. *Sep. Purif. Technol.* **2020**, *240*, 116627, doi:10.1016/J.SEPPUR.2020.116627.
14. Wang, G.; Zeng, Z.; Wu, X.; Ren, T.; Han, J.; Xue, Q. Three-dimensional structured sponge with high oil wettability for the clean-up of oil contaminations and separation of oil–water mixtures. *Polym. Chem.* **2014**, *5*, 5942–5948, doi:10.1039/C4PY00552
15. Khosravi, M.; Azizian, S. Fabrication of an Oil Spill Collector Package by Using Polyurethane Foam Wrapped with Superhydrophobic ZnO Microrods/Carbon Cloth. *Chempluschem* **2018**, *83*, 455–462, doi:10.1002/cplu.201800145.
16. Liping Liang; Yuanyuan Xue; Qian Wu; Yanyan Dong; Xu Meng Self-assembly modification of polyurethane sponge for application in oil/water separation. *RSC Adv.* **2019**, *9*, 40378–40387, doi:10.1039/C9RA05855A.
17. Gao, Z.; Zhou, S.; Zhou, Y.; Wan, H.; Zhang, C.; Yao, B.; Chen, T. Bio-inspired magnetic superhydrophobic PU-PDA-Fe3O4-Ag for effective oil-water separation and its antibacterial activity. *Colloids Surfaces A Physicochem. Eng. Asp.* **2021**, *613*, 126122, doi:10.1016/J.COLSURFA.2020.126122.
18. Kong, Z.; Wang, J.; Lu, X.; Zhu, Y.; Jiang, L.; Berlin, S.-V. In situ fastening graphene sheets into a polyurethane sponge for the highly efficient continuous cleanup of oil spills., doi:10.1007/s12274-017-1484-8.
19. Jamsaz, A.; Goharshadi, E.K.; Barras, A.; Ifires, M.; Szunerits, S.; Boukherroub, R. Magnetically driven superhydrophobic/superoleophilic graphene-based polyurethane sponge for highly efficient oil/water separation and demulsification. *Sep. Purif. Technol.* **2021**, *274*, 118931, doi:10.1016/J.SEPPUR.2021.118931.
20. Wang, J.; Wang, H.; Geng, G. Flame-retardant superhydrophobic coating derived from fly ash on polymeric foam for efficient oil/corrosive water and emulsion separation. *J. Colloid Interface Sci.* **2018**, *525*, 11–20, doi:10.1016/J.JCIS.2018.04.069.
21. Yu, T.; Halouane, F.; Mathias, D.; Barras, A.; Wang, Z.; Lv, A.; Lu, S.; Xu, W.; Meziane, D.; Tiercelin, N.; et al. Preparation of magnetic, superhydrophobic/superoleophilic polyurethane sponge: Separation of oil/water mixture and demulsification. *Chem. Eng. J.* **2020**, *384*, 123339, doi:10.1016/J.CEJ.2019.123339.
